# Neuroanatomy of *Kayentachelys aprix* and *Eileanchelys waldmani* provide insights into the early evolution of the turtle brain

**DOI:** 10.1186/s13358-025-00410-4

**Published:** 2025-11-11

**Authors:** Gabriel S. Ferreira, Serjoscha W. Evers

**Affiliations:** 1 Senckenberg Centre for Human Evolution and Palaeoenviroment (HEP) at the University of Tübingen, Hölderlinstraße 12, 72074 Tübingen, Germany; 2 Department of Geosciences, University of Tübingen, Hölderlinstraße. 12, 72074 Tübingen, Germany; 3 Department of Geosciences, University of Fribourg, Chemin du Musée 6, 1700 Fribourg, Switzerland; 4 SNSB - Urwelt-Museum Oberfranken, Kanzleistraße 1, 95444 Bayreuth, Germany

**Keywords:** Testudinata, Palaeoneuroanatomy, Brain endocast, Olfactory evolution, Inner ear

## Abstract

Even though many early stem turtles are known from relatively well-preserved skulls, their neuroanatomy remains poorly understood, limiting insights into key cranial and ecological transitions. Here we reconstruct the brain, nerves, inner ears, olfactory endocasts and arteries of two early stem turtles—the Early Jurassic *Kayentachelys aprix* and the Middle Jurassic *Eileanchelys waldmani*—based on high-resolution imaging. These species document key phases of turtle cranial evolution. Our analysis documents intermediate conditions of Jurassic mesochelydians between earlier Triassic stem turtles such as *Proganochelys quenstedtii* and crown Testudines. We show that changes in the canalis cavernosus, geniculate ganglion positioning, and braincase architecture are related to cranial stiffening in turtles. Whereas *Kayentachelys aprix* retains plesiomorphic features of Triassic testudinatans (e.g., separation of recessus scalae tympani and cavum acustico-jugulare; flat processus interfenestralis morphology) or intermediate features (e.g., cranio-quadrate space modified to short canalis cavernosus; clearly tympanic stapes but with robust morphology; reduced prootic foramen but absence of secondary braincase wall of parietal-pterygoid contact), *Eileanchelys waldmani* shows essentially ‘modern’ braincase architecture, including a ventrally inclined processus interfenestralis and a fully developed cavum tympani. Additionally, anatomical traits associated with olfaction and hearing provide insights into the paleoecology of these taxa, supporting a terrestrial lifestyle for *Kayentachelys aprix* and aquatic adaptations in *Eileanchelys waldmani*. Our study highlights the utility of neuroanatomical data in refining hypotheses of turtle cranial evolution and ecology, and underscores the importance of Jurassic stem turtles for understanding the origins of crown-group traits.

## Introduction

The fossil record of early turtles is uncommonly rich, with many taxa from the Triassic to Jurassic known from both postcranial and cranial remains. However, their palaeoecology remains a highly disputed topic. Shell (e.g., Dziomber et al., 2020; Evers et al., 2025) and limb (Joyce & Gauthier, 2004) morphology, histology (Scheyer & Sander, 2007; Scheyer et al., 2014), and biomechanics (Ferreira et al., 2024) have all been employed to tackle this issue, but controversial results arise from different proxies. In this context, neuroanatomy is presented as an additional line of evidence to help not only infer palaeobiology but also potentially contribute with phylogenetically relevant characters (e.g., Hermanson et al., 2020). Although the number of neuroanatomy studies considering or focusing on turtles has grown exponentially in the past few years (for a summary of the topic, see Ferreira et al., 2023), early stem turtles (Joyce et al., 2021) are still poorly sampled, with only four studies so far tackling the brain, inner ear and nasal endocasts of *Proganochelys quenstedtii* (Lautenschlager et al., 2018), *Naomichelys speciosa* (Paulina Carabajal et al., 2019), meiolaniids (Paulina-Carabajal et al., 2017), and *Kallokibotion bajazidi* (Martín-Jiménez et al., 2021).

Here we present an assessment of the neuroanatomy of two non-perichelydian mesochelydian turtles: *Kayentachelys aprix* Gaffney et al., 1987 from the Early Jurassic Kayenta Formation of Arizona, USA, and *Eileanchelys waldmani* Anquetin et al., 2009, from the Middle Jurassic Kilmaluag Formation of Scotland. Both of these taxa document important steps in turtle evolution. *Kayentachelys aprix* was first described as the oldest hidden-necked turtle (Cryptodira; Gaffney et al., 1987) based on the presence of characters interpreted as associated with the cryptodiran jaw closure mechanism. However, *Kayentachelys aprix* was later reinterpreted as a non-perichelydian mesochelydian turtle (Joyce, 2007; Sterli & Joyce, 2007) and subsequent phylogenetic analyses have consistently supported this result. *Eileanchelys waldmani* shares many characters with later turtles (Anquetin et al., 2009), which yields a more derived position related to *Kayentachelys aprix* (Anquetin, 2010), but it is still recovered as a non-perichelydian mesochelydian turtle. Those two taxa also represent a palaeoecological transition in the turtle lineage. *Kayentachelys aprix* is considered a terrestrial turtle based on limb proportions (Joyce & Gauthier, 2004) and shell histology (Scheyer & Sander, 2007), whereas the bone histology of *Eileanchelys waldmani* (Scheyer et al., 2014), as well as its taphonomic context (Anquetin, 2011), strongly suggests an aquatic habit, possibly supported by neuroanatomical features, particularly the size of its labyrinth compared to head size (Evers et al., 2022). By analysing the endocasts of the brain (only partially for *Eileanchelys waldmani*), inner ear, and nasal (only for *Kayentachelys aprix*) cavities, and the canals and foramina associated with cranial innervation and circulation, we provide new evidence for the paleoecology of those species and novel insights into the morphological transitions related to cranial stiffening that characterize the crown-group Testudines.

## Material and methods

We analysed the neuroanatomy of two species of the early mesochelydian turtles *Kayentachelys aprix* and *Eileanchelys waldmani*. We used micro-computed tomography (µCT) to digitize two of the best-preserved specimens of these species. TMM 43670-2 (Texas Memorial Museum, University of Texas, Austin, USA) is a crushed but almost complete skull missing only parts of the skull roof (Gaffney and Jenkins Jr, 2010; Sterli & Joyce, 2007). This is the only known specimen of *Kayentachelys aprix* that preserves the cranial cavity and otic region well (Sterli & Joyce, 2007). It was µCT scanned by Matthew Colbert at the University of Texas High-Resolution X-ray CT Facility in a NSI scanner without filter, using 120 kV, 0.165 mA, and a voxel size of 31 µm. The scan is available online (https://www.morphosource.org/concern/media/000353537). NMS G 2004.31.15 (National Museums of Scotland, Edinburgh), is an uncrushed and undeformed braincase and otic region, which is the holotype of *Eileanchelys waldmani* (Anquetin et al., 2009). It was scanned by Roger Benson at the School of Earth Sciences Xray Tomography Facility of the University of Bristol with a Nikon Metrology XT H 225 ST, using 180 kV, 166 µA, and a voxel size of 21.4 µm. The scan is available online (https://www.morphosource.org/concern/media/000354509). The volumetric datasets were converted from a 16 bit to an 8 bit TIFF stack and loaded on Amira 2023.1.1 (Thermo Fisher Scientific) for postprocessing. The segmentation of the cranial, inner ear, and other neuroanatomical-relevant cavities and canals was conducted manually using the brush and lasso tools. We generated surface models from the segmented objects with the *generate surface* function in Amira, using a smoothing extent between 2 and 5, depending on the object. Surface models of the skulls of both specimens were also segmented with Amira using the magic wand tool. All surfaces were exported as STL files and loaded into Blender 4.4.3 (http://www.blender.org) for generating 3D renderings. The tomographic datasets and STL models are available on the Morphosource repository (TMM 43670-2: Media ID 000353537; NMS G 2004.31.15: Media ID 000354509; project containing all STL models: https://www.morphosource.org/projects/000756643/).

We took a set of five measurements and three ratios of skull proportions and neuroanatomical traits from the models generated from TMM 43670-2 and NMS G 2004.31.15, as well as from additional specimens of *Proganochelys quenstedtii* (MB 1910.45.2 and SMNS 16980) and *Naomichelys speciosa* (FMNH PR273). Basicranial length (BL) is defined as the anteroposterior distance from the anterior tip of the basisphenoid (disregarding the rostrum basisphenoidale) to the posterior end of the occipital condyle. Skull width (SW) is the mediolateral distance between the lateral edges of the mandibular condyles. Brain endocast volume (BEV), labyrinth volume (LV) and olfactory endocast volume (OEV) were all measured using the function *Surface Area Volume*. The labyrinth volume was measured from both labyrinths when available (*Kayentachleys aprix*, *Eileanchelys waldmani*, and the MB specimen of *Proganochelys quenstedtii*), and the average was calculated. To calculate the ratios between olfactory endocast volume and basicranial length (OEV/BL), labyrinth volume and basicranial length (LV/BL) and skull width (LV/SW), we first took the cubic root of the volume and then divided by the linear measurement, resulting in a unitless ratio. Because the cochlear region of the endosseous labyrinth of *Eileanchelys waldmani* has a relatively large ventral projection that we interpret as a cochlear duct (see results), we measured the maximum length of the endosseous cochlear duct (ECD length; as described in Walsh et al., 2009) to estimate hearing frequencies based on Walsh et al. (2009) regression formulas. We used the mean between right and left labyrinths, and divided the mean ECD length by the skull basicranial axis length — measured as the length between the base of the occipital condyle and the anterior tip of the basisphenoid in ventral view — before log transforming it.

### Comparative *description*

#### *Kayentachelys aprix*

**Fig. 1.**
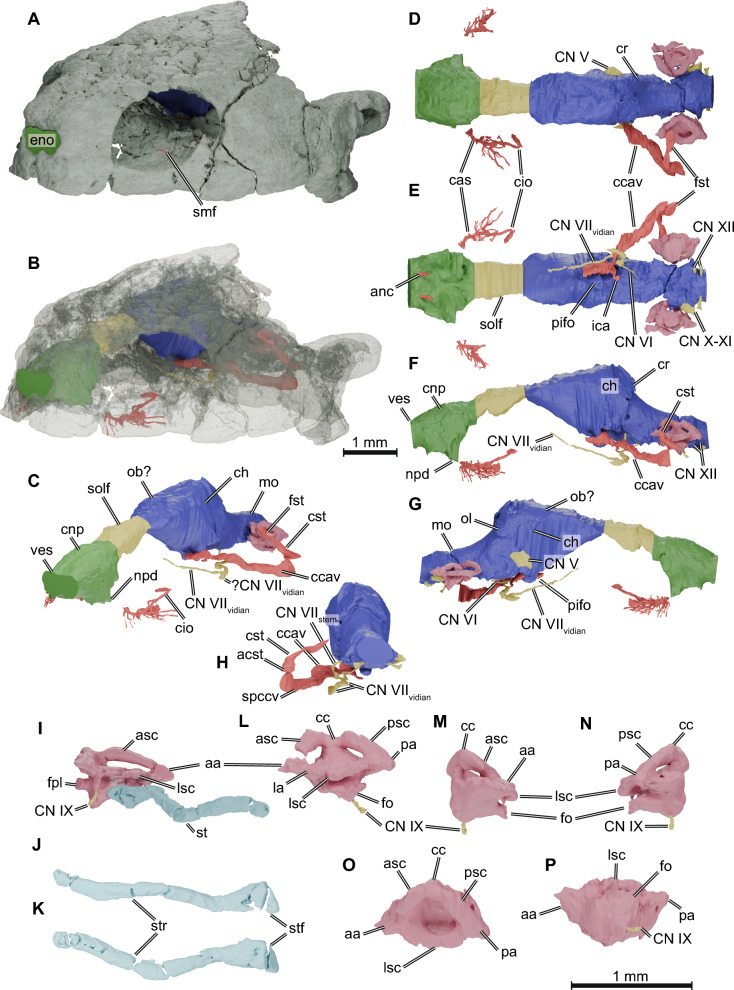
3D renderings of the skull and neuroanatomical structures of *Kayentachelys aprix* (TMM 43670-2). **A**–**C**, oblique anterolateral view of the skull (rendered solid in A, semi-transparent in **B**, and omitted in **C**) and the endocasts of the olfactory organ (green), brain (blue), endosseous labyrinth (pink), and associated cranial nerves (yellow) and arteries (red). **D**–**H**, endocranial structures in dorsal (**D**), ventral (**E**), left (**F**), right (**G**), and posterior (**H**) views. I, right stapes in posterior oblique view attached to the labyrinth and in lateral (**J**) and dorsal (**K**) views (labyrinth omitted). **L**–**P**, right endosseous labyrinth in lateral (**L**), anterior (**M**), posterior (**N**), dorsal (**O**), and ventral (**P**) views. *aa* anterior ampulla, *acst* aditus canalis stapedio-temporalis, *anc* anterior nasal artery canal, *asc* anterior semicircular canal, *cas* canalis alveolaris superior, *cc* common crus, *ccav* canalis cavernosus, *ch* cerebral hemispheres, *cio* canalis infraorbitalis, *CN V* trigeminal nerve canal, *CN VI* abducens nerve canal, *CN VII*_*vidian*_ canal of the vidian branch of the facial nerve, *CN VII*_*stem*_ canal of the facial nerve stem, *CN IX* glossopharyngeal nerve canal, *CN X-XI* common canal of the accessorio-vagus nerves, *CN XII* hypoglossal nerve canal, *cnp* cavum nasi proprium, *cr* cartilaginous rider, *cst* canalis stapedio-temporalis, *eno* external nasal opening, *fo* fenestra ovalis, *fpl* fenestra perilymphatica, *fst* foramen stapedio-temporalis, *ica* internal carotid artery canal, *la* lateral ampulla, *lsc* lateral semicircular canal, *mo* medulla oblongata, *npd* nasopharyngeal duct, *ob?* region of the olfactory bulb, *ol* optic lobe, *pifo* pituitary fossa, *pa* posterior ampulla, *psc* posterior semicircular canal, *smf* supramaxillary foramen, *solf* sulcus olfactorius, *spccv* sulcus praecanalis cavernosus, *st* stapes, *stf* stapedial footplate, *str* stapedial rod, *ves* vestibulum of the nasal cavity

*Nasal endocast*. The nasal cavity of TMM 43670-2 is completely preserved and only slightly deformed. The volume of the nasal endocast is 34% of the volume of the brain endocast. The three regions of the olfactory organ are distinguishable in the digital endocast (Fig. 1C, F): the vestibulum, anteriorly, which connects the nasal cavity to the exterior; the nasopharyngeal duct, linking the nasal cavity to the mouth roof; and the nasal cavity proper (= *cavum nasi proprium*). The short vestibulum, distinguishable from the nasal cavity only by a faint dorsal constriction, is longer than in *Proganochelys quenstedtii* (Lautenschlager et al., 2018) but shorter than in *Naomichelys speciosa* (Paulina Carabajal et al., 2019). It is surrounded by the nasal bones dorsally, the maxillae laterally, and the premaxillae ventrally. In TMM 43670-2, we identified canals (Fig. 1E) arising from the foramina prepalatina (Gaffney & Jenkins Jr, 2010), which carry the anterior nasal arteries (Albrecht, 1976) from the mouth roof anterodorsally to the vestibulum through the premaxillae. This differs from the condition of most (but not all crown turtles), in which the foramina praepalatina are simple openings not associated with canals. Here, we call these the anterior nasal canals. The nasopharyngeal ducts are short and are characterised as lateroventral projections of the nasal cavity. The ducts end at the internal nasal opening (= *apertura narium interna*), which in *Kayentachelys aprix* is bordered by the maxilla and the palatine (Gaffney & Jenkins Jr, 2010; Sterli & Joyce, 2007).

The remainder of the nasal cavity, which houses the sensorial tissue in reptiles (Parsons, 1970), is a roundish chamber roofed mostly by the frontal but also by the nasals, bounded laterally by the prefrontals and maxillae, and floored by the palatine, vomer, maxillae, and premaxillae. It shows no clear borders between dorsal and ventral portions that could be used to identify the olfactory (covered by the olfactory epithelium) and intermediate regions (which functions mainly as an airway; Parsons, 1971; Parsons & Stephens, 1968). Dorsally, the endocast of the nasal cavity shows a shallow trough (Fig. 1D), possibly associated with the cartilaginous median septum under the frontals and prefrontals, that separates the nasal cavity into two lobes (Parsons, 1959; Seydel, 1896). This septum can be partially ossified in some turtles (e.g., *Meiolania platyceps*; Paulina-Carabajal et al., 2017), marking the endocast with a deeper trough. Ventrally, the endocast exhibits a marked depression (Fig. 1E) resulting from a slightly dorsally raised midline region, formed by the premaxillae and vomer, within the nasal capsule. In *Proganochelys quenstedtii* and *Australochelys africanus* (Gaffney, 1990; Gaffney & Kitching, 1995) the vomer is strongly dorsally arched, but in later diverging turtles it becomes flat in sagittal cross-section (Anquetin et al., 2009). *Kayentachelys aprix* thus shows an intermediate condition, i.e., a slightly dorsally curved vomer. Posterodorsally, the nasal cavity transitions to the olfactory duct through which the olfactory nerve (= cranial nerve or CN I) extends. In crown-turtles (e.g., Gaffney, 1979, 1990), but also later diverging stem turtles such as paracryptodires (Evers et al., 2021 [*Arundelemys*]; Rollot et al., 2021a [*Uluops*]), the transitional area between the olfactory duct and nasal cavity is constricted by prefrontal-vomer contacts to either side of the skull midline, which usually takes the form of a keyhole and is called the fissura ethmoidalis (Gaffney, 1972). In *Kayentachelys aprix*, the fissura ethmoidalis is not yet constricted to a keyhole, and the vomer-prefrontal contact flares laterally, leaving a transversely broad opening between the nasal cavity and olfactory duct (Fig. 1D–F). The same morphology can also be seen in *Proganochelys quenstedtii* (Gaffney, 1990).

*Brain endocast*. The brain cavity of TMM 43670-2 retains a good 3D preservation for the most part; it is only broken and collapsed in some parts, particularly ventrally at the edge of the lateral braincase wall, formed by the prootic, parietal, and epipterygoid. Thus, the reconstructed morphology of the brain endocast should be interpreted more cautiously in this area (Fig. 1F, G). *Kayentachelys aprix* lacks a secondary lateral wall of the braincase, such that there is no parietal-pterygoid contact via a descending process of the parietal and ascending process of the pterygoid (crista pterygoidei), which is a derived topological contact in turtles (Ferreira et al., 2020; Gaffney, 1979; Miller et al., 2023). However, as a laterosphenoid bone is also absent, the anterolateral region of the brain of *Kayentachelys aprix*, including the trigeminal region, is largely unossified, such that this anteroventral region of the endocast should likewise not be overinterpreted. Overall, the brain endocast of TMM 43670-2 is slender and elongated, with poorly defined brain regions (Fig. 1C–G). The cephalic (between the fore- and midbrain) and pontine (between the mid- and hindbrain) flexures are pronounced (Fig. 1F).

The olfactory duct carries the olfactory nerve anteriorly from the olfactory bulbs’ region of the endocast to the posterodorsal edge of the nasal cavity, where the CN I reaches the olfactory epithelia of the olfactory organ (Fig. 1C–G). It is bounded dorsally by the frontal and laterally by the descending processes of the prefrontal (= *sulcus olfactorius*; Gaffney, 1979) and ventrally by the interorbital septum cartilage (Ferreira et al., 2023). The olfactory duct in TMM 43670-2 is broad, only slightly narrower than the remainder of the endocast, as in *Proganochelys quenstedtii*, *Kallokibotion bajazidi*, and meiolaniids (Lautenschlager et al., 2018; Martín-Jiménez et al., 2021; Paulina-Carabajal et al., 2017) but relatively short (about 15.1% of the brain endocast’s length). This contrasts with the narrower sulcus olfactorius in most perichelydian stem turtles such as paracryptodires (e.g., Evers et al., 2021) and in *Naomichelys speciosa* (Paulina Carabajal et al., 2019). A slight constriction separates the olfactory duct from the olfactory bulb region (Fig. 1D). The olfactory bulb progresses to the cerebral hemispheres without marked edges. Both brain regions have the same width, as shown by the calculated olfactory ratio (defined as the maximum width of the olfactory bulb divided by the maximum width of the cerebral hemispheres; Zelenitsky et al., 2008, 2011) of nearly 1 (OR = 0.963; Table 1). The pituitary fossa is formed as a small projection of the endocast ventral to the cerebral hemispheres (Fig. 1G). The endocast shows a strong cephalic flexure between the fore and midbrain of about 110°, but, except for this cephalic flexure, there is no well-defined transition between the cerebral hemispheres and the optic lobe posteriorly. Dorsal to the optic lobes region between the parietal and supraoccipital, there is a protuberance (Fig. 1D, F), Y-shaped in posterior view, formed by an anterodorsal midline crest and two lateroventrally directed projections that form a step towards the remainder of the optic lobes. We interpret this as the cartilaginous rider, following arguments of Werneburg et al. (2021) showing that similar morphologies are formed in extant turtles by the abrupt anterior end of the supraoccipital ossification. The pontine flexure between the mid- and hindbrain forms an angle of 125°, but, again, no clear boundaries between those regions can be identified. The hindbrain endocast is a narrow and long tube, only slightly constricted laterally at the position of the endosseous labyrinths. The region where the cerebellum is usually located has the same diameter as the medulla oblongata (Fig. 1F, G), suggesting a poorly developed cerebellum.

**Table 1:** Measurements of neuroanatomical traits in stem turtles

Taxon	Specimen	BL [mm]	SW [mm]	OEV [mm^3^]	OEV/BL	BEV [mm^3^]	LV [mm^3^]	LV/SW	LV/BL
*Kayentachelys aprix*	TMM 43670-2	22.47	56.25	603.3	0.376	1735.59	50.03	0.065	0.164
*Eileanchelys waldmani*	NMS G 2004.31.15	15.19 (18)	38.4	–	–	–	164	0.142	0.304
*Proganochelys quenstedtii*	SMNS 16980	29.86	89.73	3709.39	0.518	3790.56	–	–	–
*Proganochelys quenstedtii*	MB 1910 45 2	41.77	76.5	12,209.34	0.551	8170.84	828.86	0.123	0.225
*Naomichelys speciosa*	FMNH PR273	45.24	78.37	3881.3	0.347	8905.48	704.18	0.113	0.197
*Meoilania platyceps*	MMF 13825a	46.3	169.1	67,100	0.878	37,700	–	–	–
*Niolamia argentina*	MLP 26–40	52.9	164.8	91,600	0.852	64,900	–	–	–

*BEV* brain endocast volume, *BL* basicranial length, *LV* labyrinth volume, *OEV* olfactory endocast volume, *SW* skull width. See the methods section for descriptions of the measurements

The values for *Meiolania platyceps* and *Niolamia argentina* were retrieved from Paulina-Carabajal et al. (2017). The estimated total basicranial length (BL) of *Eileanchelys waldmani* is shown in parentheses

*Spaces associated with cranial nerves and circulation*. Although the skull TMM 43670-2 is poorly preserved on its exterior surface and somewhat crushed internally, many canals, troughs, and foramina related to blood circulation and cranial nerves can be identified in the CT data. For example, we identified and reconstructed the canalis infraorbitalis in the maxilla (Fig. 1B–G), which carries the supramaxillary artery (Albrecht, 1976). This canal, thus far unknown in other early turtles (Lautenschlager et al., 2018; Martín-Jiménez et al., 2021; Paulina Carabajal et al., 2019; Paulina-Carabajal et al., 2013), extends from the supramaxillary foramen within the floor of the orbit towards the snout. Many branches arise anterior to the canalis infraorbitalis, the main ones branch off from its lateral aspect and extend posterolaterally. The position of its anterior branching, the canalis alveolaris superior, can be identified near the anterior edge of the orbit, although its continuation is missing from the reconstructions.

**Fig. 2.**
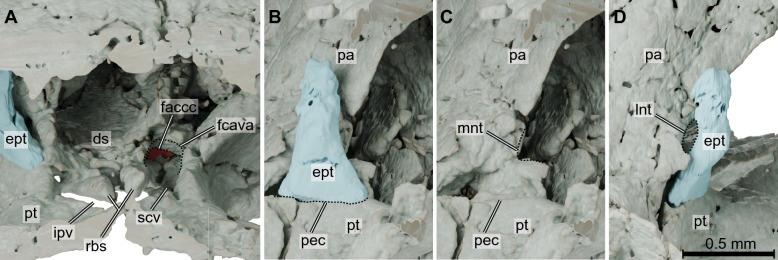
3D renderings of the foramen canalis cavernosus and trigeminal ganglion region of the *Kayentachelys aprix* (TMM 43670-2). **A**, coronally sectioned, anterodorsal view of the basicranial region focusing on the canalis cavernosus and associated structures. **B**–**C**, right anterolateral view of the trigeminal ganglion region, indicating the articulation facet of the pterygoid for the epipterygoid (dashed line in **B**) and the medial notch for the trigeminal nerve (dashed line in **C**, epipterygoid bone removed). **D**, right posterolateral view of the same region, indicating the lateral notch for the trigeminal nerve (dashed line). *ds* dorsum sellae, *ept* epipterygoid bone, *faccc* foramen anterius canalis carotici cerebralis, *fcava* foramen cavernosum anterius, *ipv* interpterygoid vacuity, *lnt* lateral notch for the trigeminal nerve, *mnt* medial notch for the trigeminal nerve, *pa* parietal bone, *pec* pterygoid-epipterygoid bone, *pt* pterygoid bone, *rbs* rostrum basisphenoidale, *scv* sulcus cavernosus

**Fig. 3.**
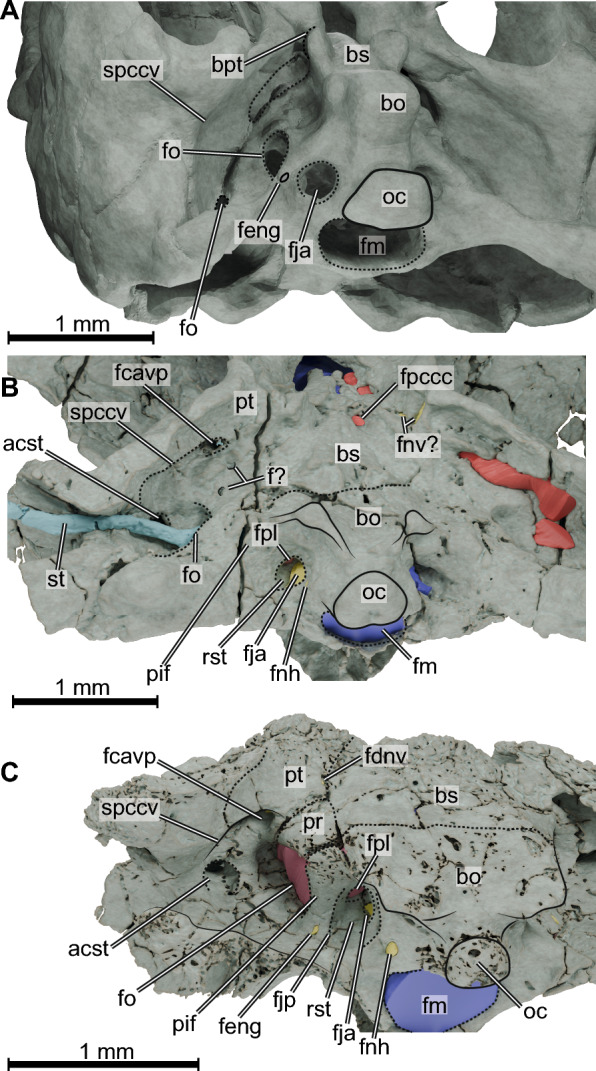
3D rendering of basicranial and occiput regions in ventrolateral views of (**A**) *Proganochelys quenstedtii* (digital model based on SMNS 16980), (**B**) *Kayentachelys aprix* (TMM 43670-2), and (**C**) *Eileanchelys waldmani* (NMS G 2004.31.15). *acst* aditus canalis stapedio-temporalis, *bpt* basipterygoid articulation, *bo* basioccipital bone, *bs* basisphenoid bone, *f?* unknown foramen, *fcavp* foramen cavernosum posterius, *fdnv* foramen distalis nervi vidiani, *feng* foramen externum nervi glossopharyngei, *fja* foramen jugulare anterius, *fm* foramen magnum, *fnh* foramen nervi hypoglossi, *fnv?* possible foramina for the vidian nerve, *fo* fenestra ovalis, *fpccc* foramen posterius canalis carotici cerebralis, *fpl* fenestra perilymphatic, *oc* occipital condyle, *pif* processus interfenestralis, *pr* prootic bone, *pt* pterygoid bone, *rst* recessus scalae tympani, *spccv* sulcus praecanalis cavernosus, *st* stapes

The canalis cavernosus extends anteriorly from its entrance between the prootic, pterygoid, and quadrate (Fig. 1C–F), which we herein call *foramen cavernosum posterius*. This canal is also bounded by the basisphenoid anteriorly and carries the lateral head vein, among other vessels and nerves. The anterior opening of the canalis cavernosus (here called *foramen cavernosum anterius*; = foramen cavernosum of Gaffney, 1972; Fig. 2A) can be observed on the left side of TMM 43670-2, formed by the pterygoid ventrally and laterally, the prootic, and the basisphenoid medially. From the foramen cavernosum anterius the lateral head vein would continue over the sulcus cavernosus, which can be seen in the pterygoid and is bounded medially by the paired rostra basisphenoidale in *Kayentachelys aprix* (Fig. 2A). The sulcus cavernosus opens to the interpterygoid vacuities medially (Fig. 2A), at which point the sulcus cannot be traced anymore. The path of the lateral head vein can be traced posterolaterally from the foramen cavernosum posterius through an excavation between the prootic and quadrate, which makes a dorsal curve at the level of the aditus canalis stapedio-temporalis. We here name this grooved osteological correlate within the roof of the cavum acustico jugulare as the sulcus praecanalis cavernosus (Fig. 3B), which can also be seen in *Eileanchelys waldmanii* (see below), although shorter (Fig. 3C). A similar excavation can be seen on the posteromedial side of the quadrate of *Proganochelys quenstedtii* (also curved posteriorly towards the aditus canalis stapedio-temporalis; Gaffney, 1990)*.* A short canal transmitting the cerebral artery (Fig. 1E) extends anterolaterally from the foramen posterius canalis carotici cerebralis (Rabi et al., 2013; = *foramen caroticum basisphenoidale* of Gaffney & Jenkins Jr, 2010; = *foramen posterius canalis carotici basisphenoidalis* of Rollot et al., 2021b) on the basisphenoid near its articulation to the pterygoid, and opens medioventrally in the canalis cavernosus as the foramen anterius canalis carotici cerebralis in the sella turcica of the basisphenoid (Fig. 2A). The opening of the cerebral artery canal within the canalis cavernosus is unusual, and it is unclear if bony walls between the cerebral canal and canalis cavernosus have simply collapsed in this region, or if the communication of these two canal systems is genuine. The canalis stapedio-temporalis (Fig. 1C–F) carries the stapedial artery in between the quadrate and prootic, from its ventral opening (= *aditus canalis stapedio-temporalis* Gaffney, 1979) inside the cavum acustico-jugulare anterodorsally (Fig. 3B), and then, at mid-length, it takes a dorsomedial turn to the foramen stapedio-temporalis, opening dorsally on the upper temporal fossa (Fig. 1C).

The only structures (canals or troughs) associated exclusively with cranial nerves that could be identified in TMM 43670-2 are those related to the olfactory (CN I, described above), trigeminal (CN V), abducens (CN VI), facial (CN VII), and hypoglossal (CN XII) nerves. The trigeminal nerve (CN V) leaves the brain laterally through an excavation between the parietal, pterygoid, and prootic. This anteriorly open prootic foramen leads posterolaterally to the cavum epiptericum, which is bordered also by the epipterygoid (Fig. 2B) and that in life should have housed the trigeminal ganglion (Evers et al., 2019). As the secondary lateral braincase wall is not yet ossified in *Kayentachelys aprix*, there is no true trigeminal foramen formed laterally to the cavum epiptericum. Further posterolaterally, a slit-like opening from the cavum epiptericum is formed between the parietal and epipterygoid. A thin canal on the basisphenoid running anteriorly from an unknown position on the ventral surface of the medulla oblongata (the posterior-most part of this canal could not be traced back in the µCT data) towards the canalis cavernosus (Fig. 1E) is here identified as the canal for the abducens cranial nerve (CN VI). Leaving from a more posterior position in the endocast, the stem of the facial nerve (CN VII) leaves the endocast from a more posterior position (Fig. 1F). At this position, there seems to be a larger cavity which could be the fossa acustico-facialis, but the region is crushed and we refrain from digitally reconstruct it. The stem of the undivided facial nerve then traverses the prootic leading to the canalis cavernosus, where the geniculate ganglion might lie. From this position, the hyomandibular branch would likely extend posteriorly through the canalis cavernosus (Fig. 1E–H) towards the cavum acustico-jugulare. The vidian branch develops a sinuous path just ventral to the position where the facial nerve stem enters the canalis cavernosus. The curves in this region are associated with ventral openings (Fig. 3B) but it is unclear whether those are actual foramina through which minor branches of the vidian nerve left the skull ventrally. Anterior to the sinuous path, the vidian nerve becomes thinner and runs anterodorsally, following the dorsal arching of the pterygoid (Fig. 1F, G). In the occiput, a small recessus scalae tympani is opened posteriorly. This opening was named foramen jugulare intermedius by Sterli and Joyce (2007), who argued that a recessus scalae tympani is absent. Inside this recess, the fenestra perilymphatica opens anterolaterally to the cavum labyrinthicum (Gaffney & Jenkins Jr, 2010; Sterli & Joyce, 2007) and anteromedially lies the foramen jugulare anterius (Sterli & Joyce, 2007), which carries the vagus (CN X) and accessory (XI) nerves, as well as the posterior cerebral vein from the cranial cavity (Gaffney, 1979). Although the recessus scalae tympani is morphologically still separated from the cavum acustico-jugulare, which is partially caused by the morphology of the processus interfenestralis of the opisthotic, the space contains the same openings as the recess of more advanced turtles, and we thus simply view it as an early morphology of the recessus scalae tympani, making a separate term as proposed by Sterli and Joyce (2007) superfluous.

*Endosseous labyrinth and stapes*. The region containing the semicircular canals and the labyrinth in TMM 43670-2 is crossed by many fractures and is slightly deformed. Nevertheless, the endosseous labyrinth is relatively well-preserved, and the digital reconstructions (especially the left one; Fig. 1L–P) allow the identification of its main traits (see also 3D model in Evers et al., 2022). The endosseous labyrinth is the ossified structure that contains a dorsal (pars superior) and a ventral (pars inferior or vestibule; Baird, 1970) part in vertebrates. The auditory system is complex, and auditory epithelia are distributed along different parts of the pars inferior (Baird, 1970; Hetherington, 2008). The vestibular system, on the other hand, is spread over both parts, being composed of the semicircular ducts and ampullae, contained in the pars superior, and the saccule within the pars inferior. The labyrinth in TMM 43670-2 is small relative to the whole skull (Evers et al., 2022) and the semicircular canals are low and short. The anterior and posterior semicircular canals are quasi-orthogonal (~ 100°), but they develop a wider angle (~ 115°) with the lateral semicircular canal. The anterior and posterior semicircular canals meet mid-length of the endosseous labyrinth to form the common crus (Fig. 1L), which is relatively low and has a small embayment between the dorsal-most aspects of the two semicircular canals. As in turtles generally, the vertical semicircular canals are nearly symmetrical (Evers et al., 2019, 2022; Ferreira et al., 2023; Lautenschlager et al., 2018; Martín-Jiménez & Pérez-García, 2021). Nevertheless, the anterior semicircular canal is slightly longer than the posterior semicircular canal and projects anterodorsally from the common crus before turning anteroventrally towards its most anterior reach (Fig. 1L). This region of the anterior semicircular canal forms an almost straight path before making a stark posterior turn where it meets the area of the anterior ampulla, which can be identified as a low bulge (Fig. 1M). Just posterior to it, a second low bulge indicates the area of the lateral ampulla (Fig. 1L) from which the lateral semicircular canal starts its path, first posterolaterally and then, at about one-fourth of its length, posteromedially. The posterior semicircular canal extends farther posteriorly than the position where the lateral semicircular canal meets its path. This is the thickest area of the posterior semicircular canal, where the posterior semicircular duct likely takes a medial turn and reaches the posterior ampulla (Evers et al., 2019). From there, the posterior semicircular canal develops a straight anteromedial and dorsal path towards the common crus. The spaces between the vestibulum and the semicircular canals are relatively large. The lateral semicircular canal creates the widest space, and the posterior semicircular canal the narrowest.

The dorsal surface of the vestibulum is rounded but not bulged dorsally, and, overall, fairly small in TMM 43670-2. The cavum labyrinthicum is completely floored by the basioccipital, prootic, and opisthotic. A small window, the fenestra perilymphatica (Fig. 3B), which should house the periotic sac in life (Gaffney, 1979), opens posteromedially into the recessus scalae tympani, which is completely separated from the cavum acustico-jugulare by the strongly ossified opisthotic (Fig. 3B). Nevertheless, a recessus scalae tympani is formed as a distinct cavity between the exoccipital and opisthotic, which medially opens via the foramen jugulare anterius (metotic foramen/embryonic metotic fissure of other reptiles; Ferreira et al., 2023; Rieppel, 1985). The lagena is part of the auditory organ and is not distinguishable from the saccule in the endosseous labyrinth, which is typical for turtles (Ferreira et al., 2023; Scheyer et al., 2022). The fenestra ovalis, which receives the stapes laterally, can be seen in the digital reconstruction in the ventral portion of the pars inferior (Fig. 1L–N). It is posteriorly displaced, but not as much as described for *Proganochelys quenstedtii* (Scheyer et al., 2022), differing from the condition of most turtles in which the fenestra ovalis is centrally placed. The stapes of TMM 43670-2 is a laterally (and slightly posteriorly) directed, long and relatively thick rod (Fig. 1I–K), very unlike the short and robust stapes of potential turtle outgroups, including *Eunotosaurus africanus*, milleretids, younginiforms, or choristoderes (Dudgeon et al., 2020; Gardner et al., 2010; Gow, 1997; Jenkins et al., 2025, 2025). Nevertheless, the stapedial rod of TMM 43670-2 is notably more robust than in crown-group turtles or even more crownwardly placed stem turtles such as paracryptodires (e.g., Spicher et al., 2024) or sichuanchelyids (Joyce et al., 2016: Fig. 1B), and thus similar to the morphology described for *Proganochelys quenstedtii* (Gaffney, 1990). Scheyer et al. (2022) documented that *Proganochelys quenstedtii* already has a properly expanded stapedial footplate medially. In TMM 43670-2, the medial end of the stapes is only slightly expanded into a stapedial footplate (Fig. 1J–K) that reaches the fenestra ovalis, but this may well be a preservation artifact. The stapedial rod extends laterally into the widely open incisura columella auris of *Kayentachelys aprix* (Gaffney & Jenkins Jr, 2010; Sterli & Joyce, 2007). From this position, it crosses the cavum acustico-jugulare before reaching the cavum tympani laterally (Fig. 3B), ending in a rounded distal surface. This lateral end of the stapes projects deeply within the cavum tympani. As the stapes is still articulated with the fenestra ovalis, this indicates that the cartilaginous extrastapes must have been short, and that a lateral articulation with a tympanic membrane must have been present. A tympanic middle ear was thus certainly present, possibly supporting the argumentation of Scheyer et al., (2022; see also Bronzati et al., 2024) who proposed this was already the case in *Proganochelys quenstedtii*, and thus contradicting the stapedial-quadrate articulation proposed by Gaffney (1990).

#### *Eileanchelys waldmani*

**Fig. 4.**
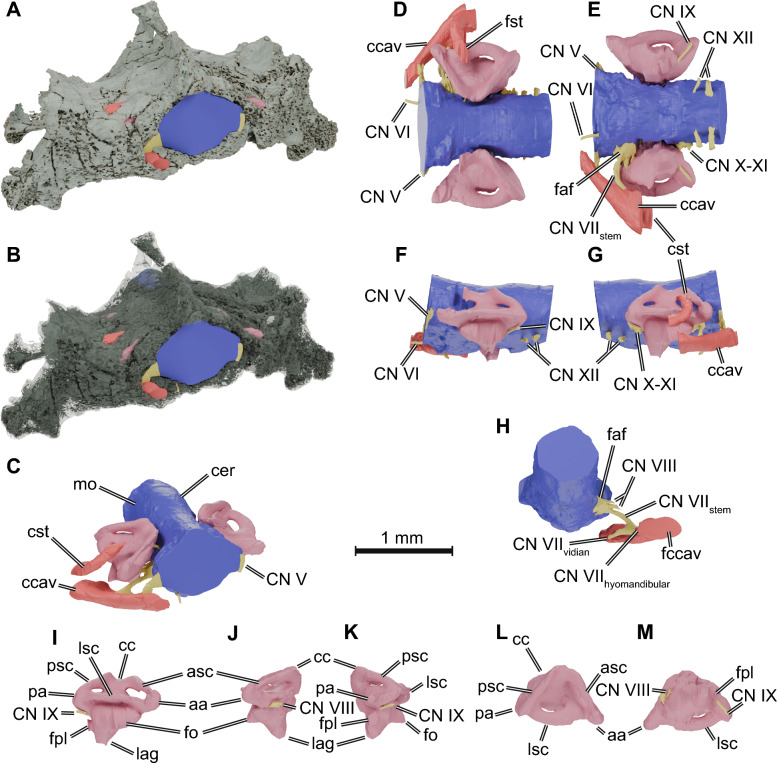
3D renderings of the skull and neuroanatomical structures of *Eileanchelys waldmani* (NMS G 2004.31.15). **A**–**C**, oblique anterolateral view of the skull (rendered solid in **A**, semi-transparent in **B**, and omitted in **C**) and the endocasts of the olfactory organ (green), brain (blue), endosseous labyrinth (pink), and associated cranial nerves (yellow) and arteries (red). **D**–**H,** endocranial structures in dorsal (**D**), ventral (**E**), left (**F**), right (**G**), and posteroventral (H) views. **I**–**M**, right endosseous labyrinth in lateral (**I**), anterior (**J**), posterior (**K**), dorsal (**L**), and ventral (M) views. *aa* anterior ampulla, *asc* anterior semicircular canal, *cc* common crus, *ccav* canalis cavernosus, *cer* cerebellum, *CN V* trigeminal nerve canal, *CN VI* abducens nerve canal, *CN VII*_*hyomandibular*_ canal of the hyomandibular branch of the facial nerve, *CN VII*_*stem*_ canal of the facial nerve stem, *CN VII*_*vidian*_ canal of the vidian branch of the facial nerve, *CN IX* glossopharyngeal nerve canal, *CN X-XI* common canal of the accessorio-vagus nerves, *CN XII* hypoglossal nerve canals, *cst* canalis stapedio-temporalis, *fo* fenestra ovalis *faf* fenestra acustico-facialis, fpl fenestra perilymphatica, *la* lateral ampulla, *lsc* lateral semicircular canal, *mo* medulla oblongata, *pa* posterior ampulla, *psc* posterior semicircular canal

*Brain endocast*. Only the braincase region of the holotype (NMS G 2004.31.15) of *Eileanchelys waldmani* is preserved, but the preservation of the inner anatomy is exquisite. Consequently, only the hindbrain could be reconstructed (Fig. 4A–G), which is described here. The hindbrain endocast of NMS G 2004.31.15 is an overall tubular structure in which the cerebellum is not distinguishable from the medulla oblongata or pons areas. At its most anterior portion, the endocast begins to expand laterally and dorsally (Fig. 4C–F) to accommodate the mid- and forebrain regions, which should therefore exhibit a greater width than the hindbrain part. At the level of the inner ears, the endocast is strongly constricted laterally (Fig. 4C), where the cranial cavity opens laterally at the hiatus acusticus. On its ventral surface, the endocast forms a pit at the suture between the basisphenoid and the basioccipital and, just posterior to it, a bulge on the body of the basioccipital (Fig. 4E). The pit in the endocast is formed by the basis tuberculi basalis (Gaffney, 1979), which serves as the attachment area for the bifid ligament of the medulla oblongata (Kesteven, 1910).

*Spaces associated with cranial nerves and circulation*. Due to the excellent preservation of NMS G 2004.31.15, many of the canals associated with cranial nerves and the blood supply of the cranium could be digitally reconstructed (Fig. 4). The morphology of the prootic anteriorly seems to indicate that a cavum epiptericum was present, suggesting the further development of an inferior process of the parietal and external trigeminal foramen. We interpret this area as the proximal portion of the trigeminal ganglion (CN V; Fig. 4C–D, F). It is important to note, however, that no specimen of *Eileanchelys waldmani* shows an exposed external trigeminal foramen or a processus inferior parietalis (Anquetin, 2010), so clarification of this trait should await further work on this taxon. Just posterior to this area lies a relatively large cavity on the medial prootic surface from which three canals exit the cavum cranii. We identify the cavity as the fossa acustico-facialis and the canals as those housing the facial (CN VII) and vestibulocochlear (CN VIII) nerves. The most anterior of those carries the facial nerve ventrolaterally towards the canalis cavernosus but bifurcates just before reaching the latter (Fig. 4H). At the bifurcation, it develops two canals, an osteological correlate for the position of the geniculate ganglion (Rollot et al., 2021a). The first, a relatively long ventral canal, the canalis pro ramo nervi vidiani, extends medioventrally and ends at the foramen distalis nervi vidiani (Rollot et al., 2018) on the pterygoid (Fig. 3C). In crown group turtles, this canal carries the vidian nerve from the facial nerve canal sensu stricto into the canalis caroticus internus (Gaffney, 1979; Rollot et al., 2021a). In NMS G 2004.31.15 and other turtles in which the carotid artery is not yet or not fully internalized (e.g., Rabi et al., 2013; Sterli et al., 2010; Rollot et al., 2018: *Eubaena*; Evers et al., 2020: *Pleurosternon*), the canalis pro ramo nervi vidiani opens ventrally on the cranium (Fig. 3C), where the vidian nerve presumably associates with the palatine artery and enters the skull more anteriorly through the openings associated with that blood vessel. The second subordinate facial nerve canal carries the hyomandibular branch of the facial nerve and is much shorter. It turns posterolaterally into the canalis cavernosus (Fig. 4H) and can be referred to as the canalis nervus hyomandibularis distalis (Rollot et al., 2021a). The other two canals associated with the fossa acustico-facialis are much shorter and reach the inner ear cavity (Fig. 4E,H), carrying two branches of the vestibulocochlear nerve (CN VIII). Turtles generally have three nerve branches of the vestibulocochlear nerve (Evers et al., 2019; Ferreira et al., 2023), such that two foramina may be either merged or collapsed into one in NMS G 2004.31.15.

Posterior to the inner ear, the paths of the cranial nerves IX to XII are visible. The proximal portion of the glossopharyngeal nerve (CN IX) exits the cranial cavity through a small foramen in the anteroventromedial part of the opisthotic that partially ossifies the hiatus acusticus. According to Gaffney (1979), this can be called the foramen medialis nervi glossopharyngei. The glossopharyngeal nerve then is confluent with the cavum labyrinthicum, until its separate distal portion pierces the processus interfenestralis (Fig. 4E, F) of the opisthotic. It exits the cavum labyrinthicum posteriorly just ventral to the posterior semicircular canal and leaves the skull through the foramen externum nervi glossopharyngei (Fig. 3C). The vagus (CN X) and accessory (CN XI) nerves leave the lateroventral aspect of the medulla oblongata region of the endocast (Fig. 4E, G) by the foramen jugulare anterius, which opens laterally into the recessus scalae tympani. Because the processus interfenestralis is ventrally deeper and more process-like in *Eileanchelys waldmani* than in *Kayentachelys aprix*, the recessus scalae tympani is deeper and better developed in the former (Fig. 3C). Finally, even further posteriorly, the two separate canals carrying branches of the hypoglossal nerve (CN XII) exit the cranial cavity ventrolaterally (Fig. 4E-G) and form two lateral foramina separated by a stout bar of the exoccipital. The most anterior of those opens laterally into the cavum acustico-jugulare, whereas the posterior foramen opens in the occiput (Fig. 3C; Evers & Benson, 2019). The orientation of hypoglossal foramina towards the cavum acustico-jugulare is rare, but has previously also been described for *Meiolania platyceps* (Evers & Benson, 2019) and *Kallokibotion bajazidi* (Evers & Benson, 2019), the latter of which has both foramina opening within the cavum acustico-jugulare.

The canalis stapedio-temporalis and the canalis cavernosus are perfectly preserved in NMS G 2004.31.15. The posterior entrances to both canals are located in the cavum acustico-jugulare, just lateral to the fenestra ovalis. The aditus canalis stapedio-temporalis is dorsal to and smaller than the entrance of the canalis cavernosus (Fig. 3C). The canalis stapedio-temporalis is relatively short and bordered along its entire length by the prootic and quadrate. It runs anteriorly but shortly after its entrance takes a 90-degree dorsomedial turn and carries the stapedial artery to the foramen stapedio-temporalis (Fig. 4C, D, G), which opens on the floor of the upper temporal fossa. The entrance to the canalis cavernosus is bordered by the quadrate laterally, the prootic medially, and the pterygoid ventrally. Just anteromedial to this entrance lies the foramen associated with the posterior canal of the hyomandibular branch of the facial nerve (CN VII; see above). In fact, the posterior end of the canalis cavernosus is medially expanded (Fig. 4C, F), and thus this branch of the facial nerve seems to have run parallel to the lateral head vein (see also Rollot et al., 2021a). The remainder of the canalis cavernosus carries the lateral head vein anteromedially to the limit of the preserved skull, at which point it also communicates dorsomedially with the cavum epiptericum (Fig. 4C–E).

*Endosseous labyrinth*. Both endosseous labyrinths of NMS G 2004.31.15 were almost perfectly reconstructed (Fig. 4), except for the anteriormost part of the left anterior semicircular canal, which is eroded in the holotype (Anquetin, 2010). The endosseous labyrinth is relatively large (see also Evers et al., 2022). The semicircular canals are oval in cross-section. The anterior and posterior semicircular canals are joined at the common crus, in which there is a distinctly marked central embayment (Fig. 4I) that is greater than in *Kayentachelys aprix*. The asymmetry of the vertical semicircular canals is more marked in NMS G 2004.31.15, as the anterior semicircular canal is distinctly longer than the posterior semicircular canal (Fig. 4L, M) and runs anteroventrolaterally along an almost straight path until it reaches an expanded area where the anterior ampulla is located. From this position, it takes a posteromedial turn and merges with the bulge of the lateral ampulla. The lateral ampulla region is less expanded than that of the anterior ampulla (Fig. 4H), and from this position, the lateral semicircular canal develops a curved path before merging with the posterior semicircular canal posteriorly. The turning of the posterior semicircular duct can be observed in the curvature of the posterior semicircular canal (Fig. 4K), and it lies ventrally to the lateral semicircular canal. From this position, the posterior semicircular canal runs along an almost straight path towards the common crus.

The pars inferior is well-ossified in NMS G 2004.31.15; the cavum labyrinthicum is almost completely floored by bone, and the area of the hiatus acusticus towards the cavum cranii is relatively small. The floor of the cavum labyrinthicum is formed by the basioccipital, prootic, and opisthotic, which develop a loose suture ventral to the position of the fenestra ovalis (Fig. 3C). The fenestra ovalis is large. It is certainly proportionally larger in comparison to the entire labyrinth size than in *Kayentachelys aprix*, but also in comparison to the braincase than in *Proganochelys quenstedtii* (Scheyer et al., 2022). This size increase is possibly associated with the different braincase architecture: in NMS G 2004.31.15 the opisthotic forms a properly ventrally directed, anatomically modern processus interfenestralis, which borders the fenestra ovalis posteriorly. The large size also likely indicates a larger stapedial footplate compared to *Kayentachelys aprix*. The fenestra ovalis of NMS G 2004.31.15 is peculiar in that its shape describes an oval (Fig. 4I), in which the anteroposterior axis is nearly twice as long as the dorsoventral axis is high. Usually, in turtles, the fenestra ovalis is round (Evers et al., 2019) bordered by the opisthotic posteriorly and the prootic anteriorly. The fenestra ovalis opens laterally into the cavum acustico-jugulare (Fig. 3C). A relatively long and conical lagena is present in the pars inferior ventral to the fenestra ovalis (Fig. 4I–K). Unlike in more crownward turtles, there is a distinct, pocket-like depression in the floor of the basicranium, formed by the opisthotic and basioccipital and positioned ventrally to the fenestra perilymphatica, which can unambiguously be identified as a “cochlear”/ “lagenar” canal. We measured *Eileanchelys waldmani* is one of the few turtles from which the cochlear/lagenar length can be quantified. Posteriorly, the periotic sac projects through the fenestra perilymphatica into the recessus scalae tympani, which is separated from the cavum acustico-jugulare by the processus interfenestralis of the opisthotic (Fig. 3C). Unfortunately, no specimen of *Eileanchelys waldmani* preserves the stapes, but the incisura columella auris is oval and wide (Anquetin, 2010) and the completely preserved quadrate indicates that a suspension of the stapes from the tympanic membrane was certainly present (see also Foth et al., 2019).

## Discussion

### Early evolution of cranial nerve canals and foramina

Our analysis of the neuroanatomy of *Kayentachelys aprix* and *Eileanchelys waldmani* reveals new information about the evolution of some traits that characterize the crown-group of turtles. One of those traits is the path of the vidian and hyomandibular branches of the facial nerve. In *Proganochelys quenstedtii* the geniculate ganglion is thought to be located inside the prootic shortly after leaving the cranial cavity (Scheyer et al., 2022). From this position, the vidian branch (also called *palatine branch*; Gaffney, 1990) would extend anteriorly through the cranioquadrate space, and the hyomandibular branch would run posteriorly in the direction of the stapes (Gaffney, 1990). In crown turtles, the proximal path is longer, and the geniculate ganglion is positioned within the canalis cavernosus in cryptodires and in pleurodires within the internal carotid canal (Gaffney, 1979; Rollot et al., 2021b). The hyomandibular branch leaves the geniculate ganglion and exits the skull posteriorly. The vidian branch continues anteriorly through the internal carotid canal, passing from the canalis cavernosus through the foramen pro ramo nervi vidiani in cryptodires. It leaves the internal carotid canal and continues to travel anteriorly through the vidian nerve canal (Albrecht, 1976; Rollot et al., 2021b).

In *Kallokibotion bajazidi*, helochelydrids, baenids, and pleurosternids (Evers et al., 2020, 2021; Joyce et al., 2014; Martín-Jiménez et al., 2021; Rollot et al., 2018, 2021a), after leaving the geniculate ganglion in the canalis cavernosus, the vidian nerve exits the skull ventrally through the foramen distalis nervi vidiani and follows the path of the palatine branch of the carotid artery, before separating from it and entering the canalis nervus vidianus (Rollot et al., 2018, 2021a). The opening of the canalis nervi facialis into the canalis cavernosus was previously identified in *Eileanchelys waldmani* (Anquetin, 2010) and here we further clarify the path of the vidian nerve. We identified a canal reaching the foramen distalis nervi vidiani in NMS G 2004.31.15 (Fig. 3C) that leaves the canalis cavernosus, showing that the vidian nerve in *Eileanchelys waldmani* followed a path similar to that of later stem turtles. Although we cannot confirm where exactly the vidian nerve reentered the skull, it is reasonable to assume it followed the carotid artery for a short distance and then entered its own canal, as is also the case in *Heckerochelys romani* (Obraztsova et al., 2024).

*Kayentachelys aprix*, on the other hand, shows a different pattern. We identified a proximal canal of the facial nerve leaving the cavum cranii through the basisphenoid and prootic and reaching the canalis cavernosus dorsomedially (Fig. 1H), as in *Eileanchelys waldmani*. Although this area is slightly crushed in TMM 43670-2, it should correspond to the position of the geniculate ganglion, as proposed by Rollot et al. (2018), because another canal branches off this area ventromedially, which we interpret as the vidian nerve canal (Fig. 1H). The hyomandibular branch likely continues posteriorly through the canalis cavernosus, but there is no clear medial trough within the latter as in *Eileanchelys waldmani*. Shortly after exiting the geniculate ganglion, the vidian canal develops a sinuous path within the contact region between the prootic and the pterygoid, where it crosses the path of the canalis cavernosus ventrally. In this area, there seem to be ventral and lateral openings that could be related to branches of the vidian nerve exiting the skull (Fig. 3B). This area is, however, somewhat crushed in TMM 43670-2 and we could not identify similar structures in other specimens of *Kayentachelys aprix* (Gaffney & Jenkins Jr, 2010; Sterli & Joyce, 2007), so those could be preservation artefacts. From this position, the vidian nerve follows a straight anterior path through the pterygoid. Thus, in *Kayentachelys aprix,* the geniculate ganglion is located more laterally than in *Proganochelys quenstedtii*. It was positioned next to the canalis cavernosus, as in later stem turtles, but, unlike those, the vidian nerve was still not associated with the palatine branch of the carotid artery and was completely encased by bone within the pterygoid in the canalis nervus vidianus. We further confirm that the foramen ventrally in the prootic identified by Sterli and Joyce (2007; Fig. 5D) and Gaffney & Jenkins Jr. (2010; Fig. 2) does not correspond to the foramen nervi facialis, as it is not connected to the above-described canals. In fact, a posterolaterally directed canal travels from this foramen to a second, smaller opening, just anterior to the fenestra ovalis (Fig. 3B), whose nature remains unknown.

The capturing of the proximal canal for the vidian nerve by the internal carotid canals might have been related to the posterior expansion of the pterygoid that happened progressively over the different turtle lineages present in the Jurassic, and that also changed the carotid circulation in testudinates (Rabi et al., 2013; Rollot et al., 2021b; Sterli & de la Fuente, 2010; Sterli et al., 2010). The posterior expansion of the pterygoid is also related to the formation of the canalis cavernosus and we can see its early steps in *Kayentachelys aprix* and *Eileanchelys waldmani* (Fig. 3). The earliest testudinates, like *Proganochelys quenstedtii*, did not possess a canalis cavernosus; the lateral head vein entered the cranioquadrate space anteriorly, through an opening formed by the pterygoid, prootic and basisphenoid (Gaffney, 1990). The distance between this opening and the entrance for the stapedial artery (canalis stapedio-temporalis in Gaffney, 1990, Fig. 27) is long. The quadrate has a curved groove on its medial aspect, which we named the sulcus praecanalis cavernosus (Fig. 3A). We interpret this sulcus as the osteological correlate of the exposed path of the lateral head vein before entering the skull. In *Kayentachelys aprix* the pterygoid is longer posteriorly, bracing the basipterygoid articulation and rendering it less mobile. As a consequence, the lateral head vein enters the skull through the foramen cavernosum anterius (Fig. 3B), formed more posteriorly than its entrance in *Proganochelys quenstedtii*, and a proper canalis cavernosus is formed anteriorly. The quadrate of *Kayentachelys aprix* also shows a well-marked sulcus praecanalis cavernosus (Fig. 3B), but shorter than in *Proganochelys quenstedtii*. *Eileanchelys waldmani* illustrates the further posterior expansion of the pterygoid, which almost completely covers the sulcus praecanalis cavernosus (Fig. 3C), shifting the entrance of the lateral head vein closer to the aditus canalis stapedio-temporalis and rendering a longer canalis cavernosus. Thus, it seems that changes related to the stiffening of the skull in turtles (Ferreira et al., 2020; Rieppel, 1993; Sterli & de la Fuente, 2010) are associated not only with the derived patterns of carotid circulation and facial nerve (Rabi et al., 2013; Rollot et al., 2021b; Sterli & de la Fuente, 2010), but also with the entrance of the lateral head vein into the skull.

The ossification of the secondary lateral wall of the braincase is thought to be related to the skull stiffening that happened in turtles as well (Ferreira et al., 2020; Sterli & de la Fuente, 2010), and it changed the framing of the trigeminal ganglion. In *Proganochelys quenstedtii*, the trigeminal nerve stem exits the cavum cranii through the prootic foramen (= *medial trigeminal foramen* of Evers et al., 2019) formed on the primary lateral wall of the braincase (Bhullar & Bever, 2009; Gaffney, 1990; Lautenschlager et al., 2018; Scheyer et al., 2022), and a trigeminal foramen in the secondary braincase wall (= *lateral trigeminal foramen* of Evers et al., 2019) has not yet evolved. In later turtles (including most Testudines), the trigeminal nerve exits the cranial cavity through the trigeminal foramen, formed on the secondary lateral wall of the braincase, whereas the prootic foramen has been reduced to an anteriorly open notch. This more lateral opening frames only the maxillary and mandibular branches of the trigeminal nerve, placing the trigeminal ganglion within the cavum cranii. Although in *Proganochelys quenstedtii* the ganglion was thus still positioned extracranially, i.e., laterally to the medial (prootic) foramen, as in other non-turtle reptiles (Evers et al., 2019), the ganglion position has not really changed, whereas the braincase architecture around the ganglion has by means of reduction of the prootic foramen and the evolution of the secondary lateral braincase wall. Evers et al. (2019) have also discussed that some later diverging turtles, such as the plesiochelyid *Plesiochelys etalloni* (Anquetin et al., 2015) and the pleurodire *Pelomedusa subrufa* (Evers & Benson, 2019), have both foramina, and the cavum epiptericum (which houses the trigeminal ganglion) is positioned between them.

*Kayentachelys aprix* shows a unique condition, in which neither medial nor lateral foramina are fully formed. A notch is formed between the prootic and the parietal (Fig. 2C), which we interpret as the homologue of the medial (prootic) foramen. Gaffney & Jenkins Jr. (2010, Fig. 12A) identified a large space between the epipterygoid and the parietal as the foramen nervi trigemini; however, after rearticulating the epipterygoid of *Kayentachelys aprix* it becomes clear that this is not an actual foramen, because the epipterygoid likely did not contact the parietal dorsally, thus forming no foramen. We identify this instead as the lateral notch of the trigeminal nerve (Fig. 2D), which should be homologous to the lateral trigeminal foramen (Evers et al., 2019). The space between those two notches is the cavum epiptericum, which in *Kayentachelys aprix* is thus partially ossified; it is bounded posteriorly by the prootic and parietal and laterally by the epipterygoid as in later turtles, but also medially by the clinoid process of the basisphenoid as in *Proganochelys quenstedtii* and *Palaeochersis talampayensis* (Gaffney, 1990; Sterli et al., 2007). Moreover, if the identification of a laterosphenoid in specimen MNA V1558 of *Kayentachelys aprix* is correct (identified as a “pleurosphenoid” by Sterli & Joyce, 2007 and Gaffney & Jenkins Jr., 2010), its pila antotica could have formed the anterior margin of a true medial foramen as in *Proganochelys quenstedtii* (Bhullar & Bever, 2009). It seems like *Heckerochelys romani* showed a condition similar to *Kayentachelys aprix*, in which both medial (visible on the prootic; Obraztsova et al., 2024; Fig. 8E-F) and lateral notches are present, although no ossified laterosphenoid has been recovered for this taxon. The condition in *Eileanchelys waldmani* remains unknown, but the morphology of the prootic suggests the presence of a cavum epiptericum. Notably, the epipterygoid of *Kayentachelys aprix* retains a plesiomorphic relationship with the braincase, which is also observed in many other reptiles: the bone has a broad base that articulates with the pterygoid, and dorsally it becomes rod-like and is suspended from the parietal/surrounding braincase bones via a soft tissue connection. This also facilitates the slight disarticulations seen in TMM 43670-2. The epipterygoids of crown turtles are commonly described as plate-like (e.g., Gaffney, 1979; Sterli et al., 2013) and are usually ventrally articulated to the pterygoid but most commonly limited to areas of the secondary lateral braincase below the (lateral) trigeminal foramen. This morphology essentially matches the ventral half of the epipterygoid of *Kayentachelys a*prix, such that we propose that the evolution of the secondary braincase wall involved a dorsal reduction of the epipterygoid. The further development of a descending process of the parietal may have rendered the presence of a cartilaginous or ossified laterosphenoid redundant and it was likely completely lost at some point in mesochelydians.

### Paleoecology of early turtles

There was a reduction in the width of the sulcus olfactorius from the Testudinata node towards the crown group. Most testudines have a narrow space between the two parasagittal ridges that separate the orbit from the sulcus olfactorius (except for *Carettochelys insculpta*; Rollot et al., 2024; some testudinids also show a broad sulcus olfactorius, but in this case it might be due to the anterior extension of the olfactory bulb, that results in a very short olfactory nerve; Paulina-Carabajal et al., 2017), but the earliest Testudinata, e.g., *Proganochelys quenstedtii* (Gaffney, 1990; Lautenschlager et al., 2018) and *Condorchelys antiqua* (Sterli et al., 2018) have wide spaces. Here, we show that this is also the case in *Kayentachelys aprix* (Fig. 1), further supporting this as a consistent trait among early testudinates. Although we do not know the condition in *Eileanchelys waldmani*, the closely related *Heckerochelys romani* already shows a narrow sulcus olfactorius (Obraztsova et al., 2024), as do *Naomichelys speciosa* (Joyce et al., 2014), paracryptodires (e.g., *Pleurosternon bullockii*; Evers et al., 2020; and *Lakotemys australodakotensis*; Rollot et al., 2022), plesiochelyids (e.g., *Plesiochelys etalloni*; Carabajal et al., 2013), and thalassochelydians (e.g., *Sandownia harrisi*; Evers & Joyce, 2020), so this state evolved certainly between the Mesochelydia and Perichelydia nodes (Joyce et al., 2021). Nevertheless, some perichelydians, such as meiolaniids (Paulina-Carabajal et al., 2017) and *Kallokibotion bajazidi* (Martín-Jiménez et al., 2021) retained the ancestral condition. Although it seems unlikely that a wider sulcus olfactorius is due only to a thicker olfactory nerve, it is noteworthy that most taxa with this trait are considered terrestrial (e.g., *Proganochelys quenstedtii*, meiolaniids, *Kallokibotion bajazidi*, and even *Kayentachelys aprix* has been suggested to be terrestrial by Scheyer et al., 2014).

The volume of the nasal cavity of *Kayentachelys aprix* is ambiguous concerning its paleoecology. As turtles lack turbinals or conchae (Martinez et al. 2024), developing larger olfactory organs is one way to attain larger surfaces covered by olfactory epithelium, thus increasing the olfactory capabilities. An enlarged cavity might additionally help thermoregulation or be used in sound production (Paulina-Carabajal et al., 2017). Because of those reasons, it has been suggested that larger olfactory organs could be associated with terrestriality in turtles (Ferreira et al., 2023; Lautenschlager et al., 2018; Paulina-Carabajal et al., 2017). The volume of the nasal cavity relative to the total endocast volume ranges from 45 to 29% in extant testudinids, but in extinct turtles it reached above 60% (e.g., in meiolaniids; Paulina-Carabajal et al., 2017). *Kayentachelys aprix* approaches the lower end of this range, with a nasal cavity about 26% of the total endocast volume. Although this does not offer strong support for a terrestrial ecology, it does not contradict it, as other taxa considered terrestrial show even lower values (e.g., in *Kallokibotion bajazidi* it corresponds to 16% of the total volume; Martín-Jiménez et al., 2021). On the other hand, the labyrinth size (Table 1) is more consistent with a terrestrial ecology for *Kayentachelys aprix*. As shown by Evers et al. (2022) terrestrial turtles tend to have relatively smaller labyrinths (although a clear functional interpretation for this relation is still unclear), whereas larger labyrinths are characteristic of aquatic and semiaquatic species. The relatively large labyrinth of *Eileanchelys waldmani* (Table 1; Evers et al., 2022) is also consistent with previous interpretations of an aquatic habit for this taxon (Anquetin et al., 2009; Scheyer et al., 2014). On the other hand, the inferred hearing range (333.74–3081,69 Hz) and best hearing frequency (2747.95 Hz) based on the endosseous cochlear duct of *Eileanchelys waldmani* are lower than those of birds, but similar to those of some crocodylians and lepidosaurs in the Walsh et al. (2009) sample. Both values are well above the hearing ranges and best hearing frequencies of *Chelydra serpentina*, *Chelonia mydas* and *Trachemys scripta* (Christensen-Dalsgaard et al., 2012; Ridgway et al., 1969; Walsh et al., 2009). Sensitivity to low hearing frequencies in these other turtles may be linked to auditory perception in aquatic environments in turtles (Christensen-Dalsgaard et al., 2012), and our results thus suggest that, although *Eileanchelys waldmani* was an aquatic species, it may have still lacked the adaptations to underwater hearing found in the crown group. However, it should be noted that the regression formulae provided by Walsh et al. (2009) are based on an overall small sample of amniotes, such that our inferred frequencies should be interpreted with caution.

In conclusion, our analysis of the neuroanatomy of *Kayentachelys aprix* and *Eileanchelys waldmani* provides new information about the evolution of cranial nerve canals and the paleoecology in early testudinates. In many aspects, *Kayentachelys aprix* still resembles earlier testudinates, such as *Proganochelys quenstedtii* (e.g., the lack of a fully formed trigeminal foramen and a vidian nerve closely associated with the internal carotid), whereas *Eileanchelys waldmani* already possesses derived traits of most braincase characters. Our results show that, already by the Middle Jurassic, the traits defining the skull morphology of modern turtles were in place, and the transitions from ancestral conditions likely happened during the Early Jurassic.

## Data Availability

The original µCT data and 3D models are available at MorphoSource. The tomographic data for TMM 43670-2 is available at https://www.morphosource.org/concern/media/000353537 and for NMS G 2004.31.15 at https://www.morphosource.org/concern/media/000354509. The 3D models are deposited in the Project ID 000756643 available at https://www.morphosource.org/projects/000756643/
